# Recent Advances across the Spectrum of Heart Failure and Heart Transplant

**DOI:** 10.3390/jcm13051427

**Published:** 2024-03-01

**Authors:** Daniele Masarone, Carlo Lombardi, Luigi Falco, Enrico Coscioni, Marco Metra

**Affiliations:** 1 Heart Failure Unit, AOS dei Colli-Monaldi Hospital, 80121 Naples, Italy; luigifalco94@libero.it; 2 Cardiology Unit, Department of Medical and Surgical Specialities, Radiological Sciences and Public Health, University of Brescia, 25121 Brescia, Italy; carlo.lombardi@unibs.it (C.L.); marco.metra@uniibs.it (M.M.); 3 Cardiac Surgery Division, AOU San Leonardo, 84100 Salerno, Italy; coscionienrico@gmail.com

## 1. Introduction

In recent years, remarkable progress has been accomplished in the heart failure (HF) landscape, with novel drugs and groundbreaking device approaches. Nevertheless, the prognosis is still severe, and patients’ quality of life (QoL) is undermined by the HF-related hospitalizations that follow from the progressive nature of the disease. Indeed, despite a variable clinical trajectory, HF relentlessly reaches the end-stage phase, for which only heart transplantation (HTx) or durable mechanical circulatory support (MCS) are viable therapeutic options. In this Special Issue, “New Advances in Pharmacologic and Non-Pharmacologic Therapy in Heart Failure and Heart Transplant”, experts in the field contributed through in-depth reviews, original research, and a network meta-analysis (NMA). We are excited to introduce 17 papers that address several topics across the HF spectrum: from pharmacological and device therapy for both chronic and advanced HF to acute HF (AHF) and its most severe form, i.e., cardiogenic shock (CS).

## 2. Chronic Heart Failure: Focus on Pharmacological Therapy

For a long time, HF therapy was limited to relieving congestion with diuretics and improving the cardiac output, reducing the afterload, and increasing contractility with vasodilators and inotropes, respectively. Subsequently, triple neurohormonal blockade and then quadruple therapy have become the standard of care (SoC). To date, several different pathways are successfully targeted with novel drugs [1,2]. Thus, HF specialists went from an era in which disease-modifying therapies were lacking to a new era, where they needed to tailor pharmacologic treatment according to patients’ phenotype. However, now physicians struggle to reach target doses and choose the proper sequence in which to introduce all recommended therapies. Indeed, when novel drugs reach phase III randomized controlled trials (RCTs), they are usually compared to a placebo on top of the SoC. Since plenty of therapies have proved effective, and head-to-head comparisons are unlikely, their respective efficacy is uncertain. In this regard, Pagnesi et al. (Contribution 1) conducted an NMA including 12 RCTs. Most of them compared Sodium Glucose Transporter 2 inhibitors (SGLT2is) to placebo, whereas two RCTs evaluated Vericiguat, and two studies randomized patients to omecamtiv mecarbil in the experimental group. SGLT2is were found to be superior to Vericiguat and omecamtiv mecarbil on the primary endpoint (a composite of cardiovascular death and HF-related hospitalizations). However, authors correctly identified differences in baseline characteristics (background use of angiotensin receptor neprylisin (ARNI), percentage of New York Heart Association (NYHA) III/IV class patients, N-Terminal Pro-B-Type Natriuretic Peptide (proBNP-NT) levels) as relevant limitations. 

With concerns for proper titration, a historical barrier is chronic kidney disease (CKD), which not only worsens the prognosis for HF and limits titration but also contraindicates the most effective therapies in advanced stages. Beltrami et al. (Contribution 2) extensively reviewed this topic and suggested a promising approach to optimize treatment in patients with a low estimated glomerular filtration rate (eGFR). In this regard, SGLT2is have demonstrated renal protection, blunting the decline of the eGFR slope, and they can even be used in stage IV CKD [3]. Sacubitril/valsartan has shown a similar effect on the eGFR slope, and in this Special Issue, Gioia et al. (Contribution 3) reported direct protective renal effects of sacubitril/valsartan, which are independent from cardiac beneficial effects. 

Another obstacle to achieving guideline-directed medical therapy (GDMT) is the progression of HF to an advanced stage. The fact that patients may gradually become intolerant to disease-modifying therapies has been extensively reported; indeed, it is included in the “I NEED HELP” criteria. In a small and selected cohort of advanced HF patients, Masarone et al. (Contribution 4) reported Levosimendan periodic ambulatory infusions as a potential enabler of the up-titration of GDMT.

## 3. Chronic Heart Failure: Focus on Device Therapy

Despite the triumphs of translational research, HF mortality is still high, comparable to many cancers. In this context, dedicated devices have become crucial to improving clinical outcomes. Besides implantable cardioverter defibrillators (ICDs) and cardiac resynchronization therapy (CRT), several new devices targeting structural abnormalities or modulating autonomic, electrophysiological, and respiratory systems are under investigation. An extensive review of valvular devices is provided by Cammalleri et al. (Contribution 5). As for autonomic modulation, growing evidence supports fluid redistribution as a critical process in the worsening of HF. Indeed, many patients do not experiment with fluid retention and weight gain before decompensation. Preclinical investigations demonstrated that the adrenergic system heavily supplies splanchnic circulation and, when stimulated, shifts a large amount of blood to the thoracic compartment. On the one hand, in healthy subjects, this is an essential mechanism to support increases in cardiac output.

On the other hand, its dysregulation in HF may contribute to a further increase in filling pressure and exercise intolerance. These concepts lead investigators to assess different approaches towards a common target: the greater splanchnic nerve (GSN) [4]. In this Special Issue, we present a pre-specified retrospective analysis of a single-arm, two-center, open-label prospective study evaluating permanent surgical ablation of the right GSN via thoracoscopic surgery in hemodynamic-adjudicated HF with preserved ejection fraction (HFpEF) patients (Surgical Resection of the Greater Splanchnic Nerve in Subjects Having Heart Failure With Preserved Ejection Fraction, NCT03715543). In this study, Gajewski et al. (Contribution 6) demonstrated that hemodynamic effects are appreciable 24 h after the procedure. As with ICD and CRT, cardiac contractility modulation (CCM) therapy is provided by leads positioned in the right ventricle. Recently, the interplay between cardiac implantable electronic devices (CIEDs) and tricuspid regurgitation (TR) has been reviewed and recognized as a distinct clinical entity that increased the risk of death [5]. Herein, a unique prospective study aimed to assess TR after CCM implantation was published by Masarone et al. (Contribution 7). Nearly half of the cohort had moderate TR, whereas patients with severe regurgitation were excluded.

Furthermore, all patients underwent CIED implantation before CCM. After six months, their TR remained stable, regardless of previous device and lead burden. The authors speculated that biventricular reverse remodeling, ventricular–arterial coupling restoration, and lowered filling pressure account for the neutral effect on TR, with possible improvements in the long term. Despite being fascinating and accurate from a pathophysiological standpoint, further studies are needed to confirm these short-term findings. In addition, CCM’s safety and efficacy were assessed, evaluating its impact on the global longitudinal strain (GLS) and mechano-energetic efficiency (MEE) for the first time. 

Finally, the AMY-CCM registry (NCT05167799) is presented. It aims to provide further insights into CCM therapy in a specific HF etiology, i.e., transthyretin amyloidosis.

## 4. Acute Heart Failure

Current guidelines identify four distinct phenotypes of AHF: acutely decompensated heart failure, acute pulmonary edema, isolated right ventricular failure, and cardiogenic shock (CS) [6]. This latter represents the most severe form of AHF, often requiring MCS. Abiragi et al. (Contribution 8) published an observational study on patients with CS who are treated by advanced HF/transplant cardiologists in a high-volume tertiary center. Unlike in most RCTs evaluating the percutaneous axial pump Impella, few patients had acute myocardial infarction (AMI). In this highly selected cohort, clinicians favored Impella in less stable patients who were post-AMI and had higher inotrope scores and body mass indexes.

Consequently, death after admission was slightly lower, albeit significant, in the Impella group. However, the overall death rates were not statistically different, and the majority of patients were successfully stabilized and even bridged to heart transplantation (HTx), highlighting the crucial role of clinicians in the selection of the more suitable MCS. Finally, a practical review summarizing recent findings on AHF management is provided by Mauro et al. (Contribution 9).

## 5. Advanced Heart Failure: Focus on Inotropes and Heart Transplantation

Although inotropes improve short-term hemodynamics, this has not translated into consistent survival benefits [7]; indeed, historical adrenergic inotropes have critical drawbacks: an increase in myocardial oxygen consumption, the trigger of arrhythmias, and stimulation of deleterious signaling pathways. Therefore, new mechanistic pathways have been explored, highlighting the pivotal role of calcium and enzymes modulating their cytoplasmatic concentration, i.e., sarcoplasmic reticulum Ca^2+^-ATPase (SERCA2a). Two comprehensive reviews summarized recent evidence on Levosimendan and Istaroxime in this Special Issue. Despite being the gold standard for advanced HF, the long-term management of HTx recipients remains complex [8]. This population has an increased thrombotic risk, and therefore, antithrombotic management is of the utmost importance. Despite direct oral anticoagulants (DOACs) becoming the first choice in several clinical scenarios due to a comparable efficacy and lower bleeding risk compared to historical anticoagulants, their use in HTx recipients is still debatable [9]. Darche et al. (Contribution 10) sought to assess the frequency, indications, and complications of DOACs and Vitamin K Antagonists (VKAs) in recipients who underwent HTx in the past 20 years. Nearly 50% of the selected cohort received a DOAC in this single-center retrospective analysis. Most patients were prescribed Apixaban or Rivaroxaban, whereas the use of Dabigatran was an exclusion criterion due to its pharmacokinetic interactions with immunosuppression agents. The VKA and DOAC groups were comparable for demographics and surgical and clinical variables. The occurrence of ischemic stroke and thromboembolic events was not statistically different between the two groups.

Conversely, the use of DOACs was linked to significantly fewer bleedings (both overall and gastrointestinal). Advances in immunosuppression regimens have improved outcomes and reduced rejection rates. However, recipients are exposed to a high infection risk. Thus, antimicrobial prophylaxis is a milestone in their comprehensive management [10]. Pneumocystis Jiroveci is an increasingly diagnosed opportunistic pathogen, especially within the first six months post HTx. Trimethoprim-sulfamethoxazole (TMP-SMX) is the first choice. Allergic or intolerant patients are usually switched to Dapsone. This latter option, however, has a less favorable safety profile than TMP-SMX. Indeed, documented glucose-6-phosphate dehydrogenase (G6PD) deficiencies may trigger hemolytic anemia. In this Special Issue, Lor et al. (Contribution 11) present a retrospective study of HTx patients who received prophylaxis with Dapsone after normal G6PD activity was documented.

Interestingly, 22% of patients developed significant anemia, and nearly 10% required hospitalization or blood transfusion. On the other hand, Dapsone withdrawal resulted in the rapid recovery of baseline hemoglobin levels. Therefore, periodic laboratory monitoring with a blood count is advisable. Besides long-term management, the main issue remains donor shortage. Hence, it is crucial to broaden the pool of potential donors to decrease the death rates among patients on the waiting list. AB0 compatibility is an essential prerequisite to HTx. Indeed, ABH antigens are expressed both on red blood cells and endothelial cells. Thus, ABO antibodies are responsible for hyperacute rejection [11]. Historically, zero patients had longer wait times, since recipients with high titers of antibodies received no organs. AB0-independent approaches were successfully implemented in kidney and liver transplantation to overcome this barrier, leading to higher transplantation rates and reduced wait times without significant safety drawbacks [12]. Limited data, however, exist for this approach in HTx. Cao et al. (Contribution 12) compared the outcomes between match and mismatch groups of patients with blood type A. The mismatch group was further divided based on donor and recipient subtypes. Due to reduced antigen expression, this has potentially critical implications, since non-A1 donors may have reduced immunogenicity, allowing for safe HTx. Although the investigators did not analyze each mismatch subgroup distinctly (i.e., non-A1 donors and A1 recipients; 44% vs. non-A1 recipients and A1 donors; 56%), the fact that significant differences in outcomes between match and mismatch groups were lacking makes it unlikely that worse outcomes among non-A1 recipients will be found.
